# Development of nutrition label use scale for patients of coronary heart disease and examination of its reliability and validity

**DOI:** 10.3389/fpsyg.2023.1168951

**Published:** 2023-10-11

**Authors:** Lu Pan, Caixia Xie, Mengjiao Liu

**Affiliations:** ^1^Clinical Nursing Teaching and Research Section, The Second Xiangya Hospital, Central South University, Changsha, China; ^2^Department of Cardiovascular Medicine, The Second Xiangya Hospital, Central South University, Changsha, China

**Keywords:** nutrition label, scale, coronary heart diseases, theory of planned behavior, intention

## Abstract

**Background:**

A proper evaluation on the intention of using nutrition label in patients with coronary heart disease (CHD) is crucial to design and formulate of behavior-based interventions. A valid and reliable instrument based on theoretical basis is needed to measure individual intention toward nutrition label use and identify underlying socio-cognitive factors.

**Object:**

To develop and test validity and reliability of the theoretically based nutrition label use (NLU) scale and to promote the use of nutrition labels in CHD patients.

**Methods:**

A questionnaire was developed based on the theory of planned behavior (TPB), empirical literatures, expert review and pilot tested. A total of 460 CHD patients in a hospital in Changsha were investigated using this questionnaire from April 2021 to August 2021. The items and dimensions in the scale were explored and confirmed using item-analysis, content validity, exploratory factor analytical (EFA), confirmatory factor analytical (CFA), internal consistency and split-half reliability tests.

**Results:**

A total of 33 items with 4 structural factors were identified, including 10 items of attitude, 6 items of subjective norm, 12 items of perceived behavior control, and 5 items of intention. The total variance explained by the EFA model was 68.563%. The model was further tested with CFA. The measurement model fitted the data well (Ratio of chi-square minimum and degree of freedom (CMIN/DF) =1.743, goodness of fit index (GFI) =0.814, incremental fit index (IFI) =0.946, Tuker-Lewis index (TLI) =0.940, the comparative fit index (CFI) =0.945, the root mean square error of approximation (RMSEA) =0.057). The content validation index (CVI) of the scale was 0.82, and the CVI of the items ranged from 0.8 to 1.00. The reliability of the scale was 0.976 (*p* < 0 0.001) using Cronbach’s alpha and 0.937 (*p* < 0.001) using the split-half coefficient.

**Conclusion:**

The newly developed Nutrition Label Use Scale can serve as a valid and reliable tool to evaluate the nutrition label use of CHD patients.

## 1. Introduction

Coronary heart disease (CHD) caused by atherosclerosis remains the leading cause of global death, accounting for 16% of all-cause death (World Health Organization, 2022). Compared with other countries worldwide, China has accounted for the largest proportion of CHD mortality in the past thirty decades, which was approximately 38.2%, posing heavy burden to individuals and families due to the repeated symptoms and frequent hospitalization (Dai et al., 2022). Among the CHD-related risk factors, unhealthy diet ranks the first (69.2%), followed by hypertension (54.4%), elevated low density lipoprotein cholesterol (41.9%), hyperglycemia (25.5%), smoking (20.6%), overweight or obesity (17.6%). These risk factors share a close association with diet, and can be positively controlled by diet management, thus delaying the progression and recurrence of CHD (Malakar et al., 2019). Moreover, diet management is considered as not only the most controllable and variable factor for individuals, but also the most effective strategy for primary and secondary prevention of CHD (Yoneda et al., 2021).

The World Health Organization (WHO) recommends the use of nutrition label as an important means of diet management (World Health Organization, 2019). Nutrition label can accurately and directly present the important nutrition information of food, which is conducive to food comparison and rapid selection for health diet (Crockett et al., 2018). Numerous studies have shown that nutrition label use is closely related to the selection of healthy food (Crockett et al., 2018), the formation of good eating habits (Talati et al., 2019), and the prevention of metabolic syndrome (Egnell et al., 2019). Moreover, intervention of nutrition label use shows favorable cost-effectiveness due to low expenditure and high benefit, making it an important means of chronic disease control and prevention, especially CHD (Du et al., 2021). Many scholars have pursued the impact, current situation analysis and intervention program of nutrition label use in various populations (Ma and Zhuang, 2021; Penzavecchia et al., 2022). A number of cross-sectional studies have also been carried out in China in recent years (Zhao et al., 2019). However, the evaluation of nutrition label use in previous studies were limited to a single aspect through self-made questionnaires without strict procedures and verification tests (Berhaupt-Glickstein et al., 2019; Shahrabani, 2021). For example, Shahrabani (2021) evaluated the perceived benefits, perceived barriers, perceived susceptibility, perceived severity and frequency of using nutrition label with a 5 points Likert scale(1 = not at all,5 = always). The scale was independently developed by the author and administered with 50 individuals for pilot test, and then the final version was formed after modification (Shahrabani, 2021). Poor reports on the psychometric properties of new or existing scales undermined the credibility of these research findings (Berhaupt-Glickstein et al., 2019; Shahrabani, 2021). Notably, the lack of instruments with systematic development process and validation will impede the design of effective interventions to successfully modify individual’s behavior. Therefore, it is essential to verify and quantify the nutrition label use scale, and establish the new scale on the basis of most suitable behavioral science model.

The theoretical foundation of behavioral science models is conducive to understand and elucidate the individual’s behavior of nutrition label use. Despite the previous application of behavioral science theory in examining factors associated with nutrition label use, few studies have used a well-structured theory to construct scales. In the study conducted by Shahrabani (2021), a questionnaire based on the Health Belief Model was used to examine the factors affecting nutrition labels. However, only the Cronbach’s alpha coefficients of two subscales were reported, not other necessary reliability and validity. Similarly, Lim et al. (2015) and Vijaykumar et al. (2013) examined factors of nutrition label use based on TPB. However, the questionnaires were developed based on literature reviews or responses from the pilot study, without important psychometric properties of the measures. Therefore, to maximize the utility of the behavioral science models on nutrition label use, it is crucial to develop a valid instrument completely aligns with the theory, which can correctly and comprehensively measure the theory constructs. Theory of Planned Behavior (TPB) (Francis et al., 2004), well-known for its ability to predict social and health behaviors (Bosnjak et al., 2020), points out that behavior is mainly determined by individual intention to implement behavior. Intention is composed of three structures: attitude, subjective norm, and perceived behavioral control (Francis et al., 2004). Rigorously designed tools are required to understand individual intention toward nutrition label use and identify underlying socio-cognitive factors, the present paper aims to develop and confirm the psychometric properties of the TPB-based instrument “Nutrition Label Use (NLU) scale.” The study attaches high importance to CHD patients who need diet management, since they are most likely to benefit from nutrition label intervention.

## 2. Materials and methods

### 2.1. Study design

A psychometric study is conducted to develop and validate a self-administered scale, so as to elucidate the use of nutrition labels in CHD patients. This study was approved by the Ethics Committee of the Second Xiangya Hospital. Ethics Approval no (2020) and Lunshen no. (Yan649).

### 2.2. Theoretical framework

The construction of the NLU scale is based on the TPB. According to TPB (Bosnjak et al., 2020), behavioral intention is considered as the tendency and necessary process of an individual to take certain behaviors, which can directly predict their actual behaviors. Intention is determined by attitudes, subjective norms (SN) and perceived behavior control (PBC). Attitude refers to an individual’s positive or negative evaluation of behavior, SN refers to the perceived social pressure when performing the behavior, and PBC refers to an individual’s perceived ability to perform the behavior. In general, a more positive attitude, stronger support from others and stronger perceived behavioral control can contribute to stronger behavioral intentions. In this study, the target behavior investigated is nutrition label use in CHD patients.

### 2.3. Development of nutrition label use scale

The procedure for forming the NLU scale was as follows. First, members of the research group obtained relevant literature by searching keywords such as “coronary heart disease” “nutrition label” and “theory of planned behavior,” and discussed and refined the literature contents to form 55 items and 4 hypothesized domains. The four hypothesized domains consisted of 22, 9, 20 and 4 items for each domain. Second, the purposive sampling method was used to select 15 experts (with experience in nursing management, nutrition management, cardiovascular diseases nursing, psychometric studies, and questionnaire development) to evaluate the scale items. The expert group received an e-mail inquiry form from the research group, and was required to score the importance of each item and put forward comments for modification. The importance score ranged from 1 to 5 points, the higher the score, the higher the matching degree between the item and the scale. After the first round of consultation, 5 items were modified and 1 item was added based on expert opinions. Besides, 17 items were removed because the coefficient of variation of their importance scores over 0.3 or a mean importance score less than 4 points. Finally, the NLU scale was trimmed down from 55 to 39 items, and the retained 39 items were sent out to the same expert panel for re-evaluation. In the light of these expert opinions, no further items were deleted.

### 2.4. Pilot testing

In March 2021, 30 hospitalized CHD patients (13 males and 17 females, average age 60.23 years) were conveniently sampled from a hospital in Changsha City for the pilot test of the 39-item NLU scale. Each item on the scale was scored on a Likert 5 points. These patients were required to comment on the clarity and comprehensibility of the scale items, the rationality of the item order and the length of time it took them to complete the scale. According to the feedback from pilot participants, the question wording for three item was modified to improve clarity and no item was deleted.

### 2.5. Participants and sampling

At the formal investigation stage, another group of hospitalized CHD patients was conveniently sampled in a hospital in Changsha City between April 2021 and August 2021. The inclusion criteria were 18 years of age or older, normal consciousness and intelligence, and certain ability to read and write. The exclusion criteria were patients with severe complications such as heart failure. All recruited respondents were informed of the purpose of this study that their participation was voluntary and anonymous, and signed an informed consent prior to filling in the NLU scale. This version of the NLU scale contains 39 items. In addition, the sample size was required to be 5–10 times of the number of items (Minglong, 2010), and an invalid recovery rate was considered to be 15%. Therefore, the sample size was calculated to be 225–449 cases.

### 2.6. Data collection

All questionnaires were distributed one-on-one by the researchers, filled out on the spot by the participants and then recycled. In order to maintain consistency and avoid influencing respondents’ answers, participants were instructed to answer the questions in the way they understood, without providing verbal explanations. After collecting the questionnaire, the researchers checked filling quality. If items were omitted or filled in regularly, the patient was requested to complete or fill in again. Finally, 500 questionnaires were distributed in this study, and 460 valid questionnaires were collected, with an effective recovery rate of 92%. No incentives were offered to complete the survey.

### 2.7. Data analysis

The data were entered and checked by two researchers and analyzed using Statistical Package for the Social Sciences (SPSS) and Advanced Mortar System (AMOS), Version 23. For all analysis, a value of *p* of <0.05 (two-tailed) was considered statistically significant.

#### 2.7.1. Item analysis

Item analysis were used in this study for further scale modifications. Item–total correlation (ITC), inter-items correlation, and Cronbach’s alpha-if-item deleted were examined through SPSS to determine which item should be retained or deleted from the scale. ITC assessed the relationship between each item and the overall scale, while inter-items evaluated the relationship between two items. Item-total correlations and inter-items correlations were calculated by Pearson’s correlation coefficient (Norušis, 1997). Cronbach’s alpha-if-item deleted reflects the difference in score reliability (Norušis, 1997). Item with an ITC less than 0.4 or inter-items correlation over 0.8 was considered to be deleted. If the Cronbach’s alpha-if-item deleted was increased, then the item was considered to be deleted.

#### 2.7.2. Content validity

Content validity refers to whether the measured content is suitable for the measurement target, which is generally determined by the consensus of experts in the field. The scorer reliability of two rounds of expert were measured by Kendall Concordance Coefficient through SPSS. The closer the Kendall Concordance Coefficient is to 1, the higher the scorer reliability. The content validation index (CVI) includes item-level CVI (I-CVI) and scale-level CVI (S-CVI), which are calculated based on the importance rating of each item of the scale from the second round expert consultant (Almanasreh et al., 2019). I-CVI > 0.78 and S-CVI > 0.8 were considered satisfactory, indicating sounds content validity (Almanasreh et al., 2019).

#### 2.7.3. Exploratory factor analysis

EFA was conducted through SPSS to identify and refine its underlying dimensions of the NLU scale, and to provide construct validity of the scale. A total of 460 questionnaires were divided into two groups according to the odd and even numbers in the sequence of retrieval, with 230 questionnaires in each group. The odd-numbered data were used for EFA, and even-numbered data for confirmatory factor analysis. Firstly, according to the Kaiser-Meyer-Olkin (KMO) measure and Bartlett’s sphericity test, the suitability of the data for EFA was determined. A KMO value more than 0.6 and a significant Bartlett’s test of sphericity were required for EFA (Osborne and Costello, 2009). Communalities were then inspected to determine suitability of each item for EFA. Each item must have a communality value greater than 0.5 or otherwise the items were excluded from EFA (Osborne and Costello, 2009). Finally, the principal component analysis with maximum variance rotation was performed for the remaining items to extract the major contributing factors. Factor extraction standards were an eigenvalue greater than 1, scree plot, cumulative percent of variance extracted greater than 50%, and the number of items included over 3 (Osborne and Costello, 2009). As suggested by Osborne and Costello (2009), items with loadings less than 0.5 in all factors or having loadings over 0.5 on two or more factors were excluded.

#### 2.7.4. Confirmatory factor analysis

Furthermore, CFA was conducted on 230 even numbered data using AMOS 23.0 to assess the goodness of fit of the EFA-derived model of the NLU Scale. Model fit was evaluated using a combination of fit indices by the following criteria: the minimum chi-square to degree of freedom ratio (CMIN/DF) ranging from 1 to 3 indicates perfect fit. Goodness of fit index (GFI), incremental fit index (IFI), Tuker-Lewis index (TLI) and the comparative fit index (CFI) greater than 0.9 indicate perfect fit or greater than 0.8 indicate reasonable good fit. The root mean square error of approximation (RMSEA) less than 0.05 suggests perfect fit or less than 0.08 suggests fairly good fit (Brown, 2006).

#### 2.7.5. Reliability

Reliability analysis was conducted by computing Cronbach’s alpha and split-half reliabilities. Cronbach’s alpha coefficient greater than 0.7 and split-half coefficient greater than 0.8 were considered to be sufficiently reliable (Minglong, 2010)^.^

## 3. Results

### 3.1. Demographic characteristics of participants

A sample of 230 CHD patients for EFA was drawn from the total sample, leaving the remaining 230 CHD patients for CFA. The mean ages of EFA sample and CFA sample were (59.76 ± 8.30) and (60.02 ± 8.559) years, respectively. The difference was not statistically significant (*t* = −3.32, *p* = 0.740). And other demographic characteristics were summarized in Table 1, without significant differences between EFA and CFA samples (see Table 1).

**Table 1:** Demographic characteristics for participants in the EFA and CFA.

Demographic characteristics	EFA sample (*n* = 230)	CFA sample (*n* = 230)	*X* ^2^	*p*
**Sex [*n* (%)]**			2.524	0.112
Men	99(43)	116(50.4)		
Women	131(57)	114(49.6)		
**Marital status [*n* (%)]**			4.859	0.182
Single	1(0.4)	0(0)		
Married	214(93)	207(90.0)		
Divorced	2(0.9)	8(3.5)		
Widowed	13(5.7)	15(6.5)		
***Per capita* income [*n* (%)]**			0.610	0.737
Low(<RMB1,3,389)	61(26.5)	67(29.1)		
Middle(RMB1,3,389–6,5,393)	128(55.7.)	127(55.2)		
High (>RMB6,5,393)	41(17.8)	36(15.7)		
**Education [*n* (%)]**			1.257	0.533
<High school	116(50.4)	125(54.3)		
High school graduate	75(32.6)	64(27.8)		
>High school	39(17.0)	41(17.8)		
**Smoking [*n* (%)]**			0.771	0.680
Never	151(65.7)	142(61.7)		
Smoking cessation	38(16.5)	43(18.7)		
Current smokers	41(17.8)	45(19.6)		
**Drinking [*n* (%)]**			2.801	0.246
Never	187(81.3)	173(75.2)		
Stop drinking	21(9.1)	31(13.5)		
Current drinkers	22(9.6)	26(11.3)		
**Course of CHD [*n* (%)]**			5.180	0.159
<1 years	85(37.0)	67(29.1)		
1–5 years	62(27.0)	82(35.7)		
6–10 years	46(20.0)	48(20.9)		
>10 years	37(16.1)	33(14.3)		

### 3.2. Item analysis results of the nutrition label use scale

The inter-items correlation coefficient between PBC10 and PBC11 was 0.830, indicating a high correlation between the two. PBC10 was deleted after discussion by the research group, and the correlation coefficients of the remaining items were acceptable (0.202 to 0.797). Item–total correlations ranged from 0.492 to 0.893, all greater than 0.4. Cronbach’s α of the scale was 0.978, without increase after deleting any item, so the remaining items were considered satisfactory. Therefore, only one item was dropped from the scale through the item analysis methods, leaving 38 items for subsequent psychometric testing.

### 3.3. Content validity results of the nutrition label use scale

Kendall Concordance coefficients of the first and second rounds of Delphin consultation were 0.256 and 0.503 (*p* < 0.001) respectively, suggesting that experts’ opinions tended to be consistent with favorable scorer reliability. The I-CVI for the nutrition label use scale ranged from 0.8 to 1.00. The S-CVI for the nutrition label use scale was 0.82. These computed CVIs were considered satisfactory (Almanasreh et al., 2019).

### 3.4. EFA results of the nutrition label use scale

The KMO value of the NLU scale was 0.969, and the Bartlett’s sphericity test exhibited significant difference (*p* < 0.001), indicating the suitability of the scale for factor analysis. Three items (AT18, AT19 and AT21) were dropped from the scale prior to factor analysis, since their communalities were 0.495, 0.380 and 0.407, respectively, lower than the acceptable limit of 0.5. The remaining 35 items were conducted with EFA to explore the domain. Four factors were produced with eigenvalues over 1 and cumulatively explained 68.56% of the variance. The variance value of each factor was 20.495, 19.100, 14.826 and 14.142, respectively (Table 2). The loadings of two items (A17 and PBC5) in four factors were less than 0.50, and the 2 items were deleted. As a result, 33 items in four factors were retained in the final model.

**Table 2:** Results of Exploratory Factor Analysis, and Reliability Analysis for EFA Sample (*n* = 230).

No. Item content	Factor loadings			
	1 AT	2 PBC	3 IN	4SN
AT1 Nutrition labels are accurate and reliable	**0.642**	0.214	0.393	0.316
AT2 Nutrition label use can help me obtain important nutrition information of food	**0.662**	0.181	0.298	0.343
AT5 Nutrition label use can help me choose healthy food	**0.683**	0.209	0.347	0.285
AT6 Nutrition label use can help me compare food	**0.720**	0.179	0.134	0.119
AT8 Nutrition label use can help me avoid eating foods with high fat and salt	**0.659**	0.232	0.232	0.014
AT9 Nutrition label use can help prevent disease	**0.600**	0.360	0.367	0.304
AT10 Nutrition label use can help in the treatment of diseases such as coronary heart disease	**0.554**	0.384	0.214	0.493
AT13 Nutrition label use promotes healthy eating habits	**0.545**	0.246	0.489	0.315
AT14 Nutrition label use is convenient	**0.713**	0.222	0.264	0.289
AT16 Nutrition label use is necessary	**0.634**	0.235	0.390	0.322
AT17 Nutrition label use makes food selection complicated	0.449	0.285	0.329	0.394
SN1 My family expect me to use nutrition labels	0.420	0.335	0.286	**0.586**
SN2 Doctors or nurses expect me to use nutrition labels	0.419	0.356	0.254	**0.611**
SN3 My friends expect me to use nutrition labels	0.195	0.200	0.415	**0.607**
SN6 Mass media(TV, newspapers, books or mobile phones) expect us to use nutrition labels	0.241	0.165	0.380	**0.622**
SN8 Fellow sufferers expect me to use nutrition labels	0.460	0.342	0.015	**0.623**
SN9 Community workers expect me to use nutrition labels	0.083	0.318	0.155	**0.692**
PBC1 The information of ingredients on nutrition labels encourage me to use it	0.486	**0.600**	0.197	0.250
PBC2 The intuitiveness of the nutrition label encourage me to use it	0.487	**0.601**	0.195	0.215
PBC3 The quest to be healthy motivates me to use nutrition labels	0.430	**0.627**	0.189	0.324
PBC5 Past experience encourages me to use nutrition labels	0.303	0.381	0.496	0.390
PBC6 Nutrition labels on the back of the food package discourage me from using it	0.232	**0.561**	0.444	0.353
PBC7 The numerical expression of the nutrition label discourage me from using it	0.389	**0.541**	0.339	0.093
PBC8 The small font size of the nutrition labels discourage me from using it	0.364	**0.514**	0.416	−0.048
PBC11 Lack of knowledge about nutrition labels discourage me to use nutrition labels	0.291	**0.589**	0.401	0.410
PBC12 Impulse eating habits discourage me to use nutrition labels	0.196	**0.778**	0.206	0.187
PBC13 Time constraints discourage me to use nutrition labels	0.109	**0.655**	0.224	0.365
PBC15 Being with someone else restricts my use of nutrition labels	0.131	**0.721**	0.174	0.242
PBC16 Preference for specific foods restricts my use of nutrition labels	0.183	**0.733**	0.182	0.250
PBC19 Family financial strain restricts my use of nutrition labels	0.385	**0.565**	0.463	0.400
IN1 I intent to learn about nutrition labels	0.364	0.272	**0.662**	0.201
IN2 I intent to use nutrition labels	0.405	0.322	**0.669**	0.315
IN3 I intent to use nutrition labels as often as possible	0.402	0.377	**0.601**	0.302
IN4 I intent to select food according to the nutrition labels	0.354	0.312	**0.722**	0.298
IN5 I intent to recommend others to use nutrition labels	0.395	0.354	**0.613**	0.287
Eigenvalue	20.037	1.755	1.205	1.000
Variance explained(%)	20.495	19.100	14.826	14.142
Cumulative variance (%)	20.495	39.595	54.421	68.563
Cronbach alpha	0.942	0.947	0.937	0.858
Split-half reliabilities	0.925	0.925	0.905	0.894

AT, attitude; SN, subjective norm; PBC, perceived behavioral control and IN, intention.

### 3.5. CFA results of the nutrition label use scale

**Figure 1:**
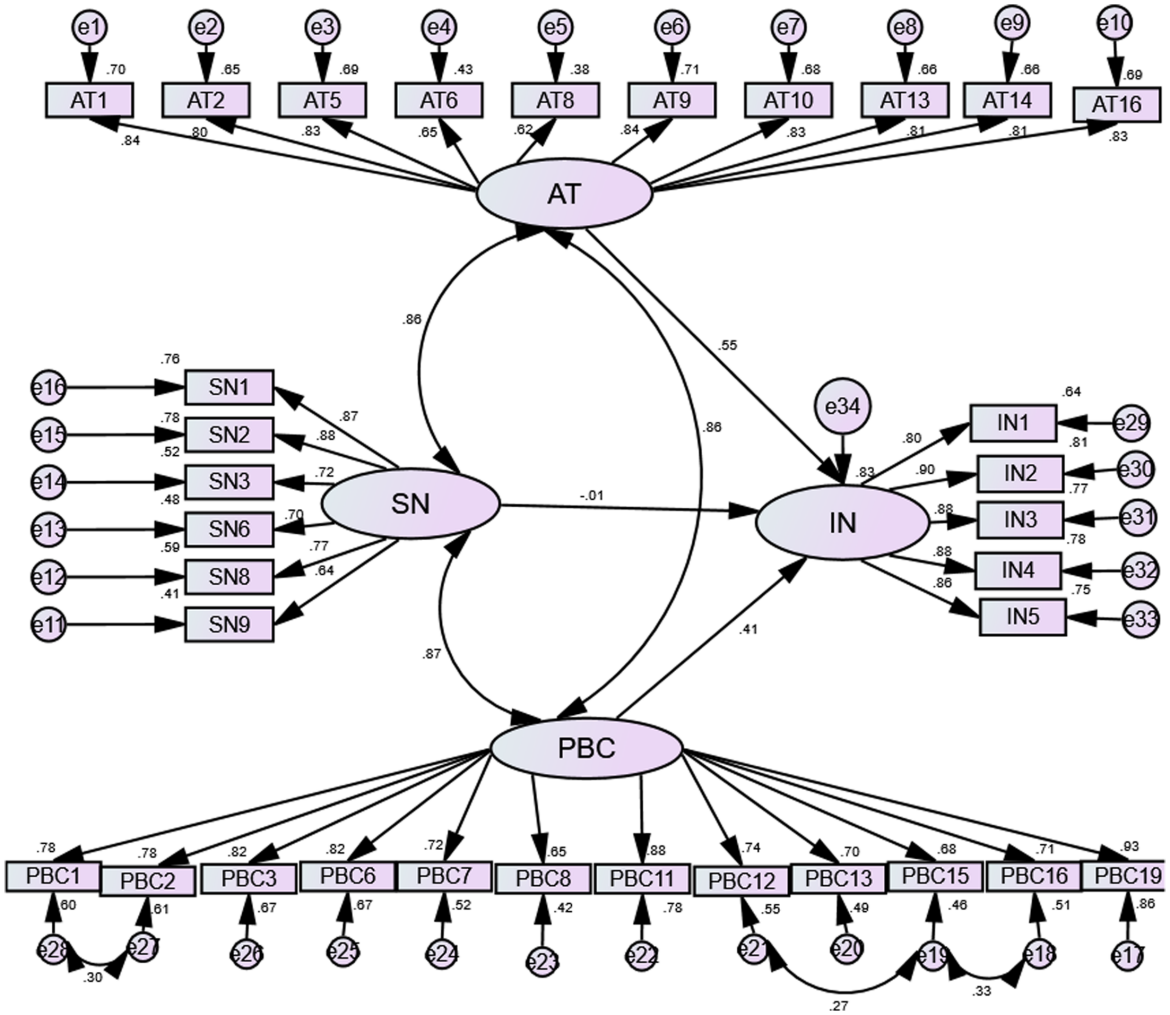
The structural equation modeling of the Nutrition Label Use scale. AT, attitude; SN, subjective norm; PBC, perceived behavioral control, and IN, intention.

The fit indices produced by CFA were within acceptable threshold values: CMIN/DF = 1.743(*p*<0.001), GFI = 0.814,IFI = 0.946, TLI = 0.940, CFI = 0.945, RMSEA = 0.057, indicating that the model was a reasonably good fit. The fitting model of CFA is shown in Figure 1.

### 3.6. Reliability results of the nutrition label use scale

Cronbach’s α coefficient of the final version NLU scale was 0.976 (*p* < 0.001), and that of each subscale was 0.942, 0.858, 0.947 and 0.937, respectively (*p* < 0.001). Likewise, the split-half reliability of the final version NLU scale was 0.937 (*p* < 0.001), and the split-half reliability of each subscale was 0.925, 0.894, 0.925 and 0.905, respectively (*p* < 0.001) (see Table 2). The results of reliability test demonstrated high reliability of the scale.

## 4. Discussion

The present study aims to develop and validate a NLU scale based on the TPB theory, so as to evaluate the intention of nutrition label use and the factors affecting intention (attitudes, SN and PBC). The NLU scale exhibited sound reliability and validity, which was evidenced by psychometric analysis. The final version of the NLU scale could be seen in Supplementary material.

The development of a nutrition label use scale is of great significance and practicability since nutrition labels are highly accessible, yet underutilized. Without evaluation instrument which completed reliability and validity tests in previous studies (Vijaykumar et al., 2013; Lim et al., 2015), researchers and clinical workers could not accurately evaluate the use of nutrition labels and provide targeted guidance and intervention. Therefore, the newly developed NLU scale in the current study figures prominently in filling this important gap. The evaluation of nutrition label use varies greatly according to research design, clinical domain, intervention strategy, target audience and follow-up time (Vijaykumar et al., 2013; Lim et al., 2015; Berhaupt-Glickstein et al., 2019; Zhao et al., 2019; Shahrabani, 2021; Penzavecchia et al., 2022). Special consideration should be given when extending the results to other nutrition label use researches or activities. Although we have developed the current scale for CHD patients with fairly homogeneous quality in the hospital, the scale can be modified to have broader utility for CHD patients at different sites, and different types of nutrition label use researches or activities. In addition, it can be served as a model and be modified to measure other target populations, such as diabetes, hyperlipidemia or obese.

One advantage of this study lies in the application of TPB to construct the NLU scale. According to DeVellis’ recommendation (DeVellis, 2016), relevant theories should be considered for the content development of the new behavioral scales. TPB is not complex, but it is widely used in various studies of healthy behavior, including the nutrition label use (Lim et al., 2015; Berhaupt-Glickstein et al., 2019). Lim et al. (2015) explored the behavioral determinants of TPB-based nutrition label use and found that the perceived behavior control had the greatest influence on nutrition label use among Korean female college students. Vijaykumar et al. (2013) investigated the factors influencing the use of nutrition labels by shoppers in Singapore based on TPB. Research results showed that age and race had significant differences in attitudes, subjective norms, and behavioral controls, among which subjective norms was significant predictors of intention to nutrition label use. Yet these measurement tools used for nutrition labels have not been validated. Therefore, an effective TPB-based instrument will further advance the field of promoting nutrition label use. Moreover, TPB can provide a framework for evaluating the intention of nutrition label use from multiple aspects (Ma and Zhuang, 2021). The results of this study showed the comprehensive content of the newly developed TPB-based NLU scale, which includes not only items such as lack of knowledge, impulse eating, time, preference for specific foods, but also evaluation of attitude, SN, PBC. Therefore, the TPB-based NLU scale sheds new light on investigation and elucidation of the occurrence, development and change of intention and even behavior itself, thereby providing great theoretical and practical value by identifying the necessary factors to modify the behavior.

In the current study, the scale was developed according to the scientific process. A multi-phase questionnaire development method was adopted, including literature reviews for gathering relevant items, expert review, pilot test, which should be standard procedures in any process of developing a survey instrument (Collins, 2003). To our knowledge, the instruments used for evaluating nutrition label in previous studies did not go through these necessary process. Lim et al. (2015) developed the questionnaire using only literature and pilot studies, while Vijaykumar et al. (2013) simply adapted and used untested items from other studies. In the present study, 15 experts were selected to review whether items represented all aspects of the area under investigation, and whether the words of the items were clear. A number of items were dropped until the experts reached an agreement. Experts had expertise in questionnaire design, social and behavioral theories, cardiovascular management, and nutrition management, with favorable representativeness and authority. The response rate of the two rounds of questionnaire consultation was 100%, indicating that the experts are highly motivated. Initial screening of items was based on two rounds of expert reviews. In addition, item analysis methods were also the basis for improving items and provides additional benefits for this study. In the current study, two items with high correlation were found which indicated redundancy after item analysis, and the item with better measurement properties was retained. Further, items were evaluated and screened through EFA, CFA and reliability test. All in all, the rigor and rationality of the scale development were ensured with the methods and processes above mentioned.

Statistical analysis displayed the sound validity and reliability of NLU scale. Validity includes content validity, construct validity and criterion-related validity. In previous studies, the most common thing in questionnaires is the lack of validity test, making it difficult for us to compare the results (Vijaykumar et al., 2013; Lim et al., 2015). Content validity describes the degree of representation between the actual content and the expected content of the scale (Rubio, 2005). Generally, it is determined by the consensus of experts in the field. In this study, the I-CVI of the NLU scale were greater or equal to 0.80, and the S-CVI was 0.82, indicating favorable content validity. Construct validity, assessed through EFA and CFA, measures the extent to which the items accurately represents the proposed construct (Rubio, 2005). Four factors that emerged from the EFA aligned well with the TPB constructs, explaining 68.563% of the variance. Each item retained was loaded with a corresponding factor of more than 0.5. Besides, the attribution of items and dimensions was basically consistent with the theoretical hypothesis. These subscales showed evidence of validity and can be confidently named as attitudes, perceived behavioral control, intention and subjective norms. Furthermore, CFA was used to test the fitting degree of the structural equation model of NLU scale, and the fitting index indicated the acceptability of the model fit, thereby achieving a sound construct validity of the NLU scale (Zhao et al., 2019) However, the criterion-related validity was not tested in this study due to the absence of classical measuring instruments for nutrition labels and the large differences in the contents and evaluation indicators of self-made questionnaires in previous literatures. Reliability describes the degree to which a test produces the same result over repeated measurements, holding other factors constant. Cronbach’s alpha and split-half reliabilities were used to test the reliability of the NLU scale, indicating good internal consistency. Our reliability estimates for the total scales and subscales were greater than 0.85, whereas the reliability estimates for the homemade nutrition label use questionnaire reported by Lim and colleagues ranged from 0.60 to 0.84 (Lim et al., 2015). Although the theory used in this study and the domains contained in the questionnaires are similar to ours, our high reliability estimates are mainly due to the rigorous scale-making process, and may be partially related to the adequate number of items included. Nunnally and Bernstein stated that “One of the major way to make the tests more reliable is to make them longer.” (Nunnally and Bernstein, 1994).

There are some possible limitations to the study. First, convenience samples of CHD inpatients were selected from a large hospital in the central China’s Hunan province. The sample was relatively homogenous in terms of culture and socioeconomic status, potentially limiting the generalizability of the results. Future studies will need to replicate these findings with different samples from diverse cultures or regions. Second, some important attributes of the scale, such as retest reliability and criterion validity, were not examined in this study, which need to be tested in the future research. Lastly, the study was cross-sectional, and utilized intention as a proxy for actual behavior of nutrition label use, which was reasonable given that the main focus of the current study was on scale development, and was also common in TPB. Nevertheless, future research can be conducted with a longitudinal design to assess actual behavior of nutrition label.

## 5. Conclusion

The newly developed Nutrition Label Use scale consist of 33 items in 4 domains, which is consistent with the structure of the TPB framework: attitude, subjective norm, perceived behavioral control and intention. The NLU scale was proved to be valid and reliable, which can be widely used in quantitative studies to assess the intention of nutrition label use and predict the actual use behavior of nutrition label. Additionally, this scale is conducive to comprehensively understanding the various factors affecting the intention of nutrition label use, and extending to the design of effective intervention programs.

## Data availability statement

The raw data supporting the conclusions of this article will be made available by the authors, without undue reservation.

## Ethics statement

The studies involving humans were approved by Ethics Committee of the Second Xiangya Hospital. The studies were conducted in accordance with the local legislation and institutional requirements. Written informed consent for participation in this study was provided by the participants' legal guardians/next of kin.

## Author contributions

LP: conceptualization, methodology, formal analysis, investigation, writing - original draft, writing—review and editing, project administration. CX: conceptualization, methodology, project administration, writing - review. ML: methodology, formal analysis, investigation. All authors contributed to the article and approved the submitted version.
