# Supplementary material for: Development of nutrition label use scale for patients of coronary heart disease and examination of its reliability and validity
Source: Front Psychol. 2023 Oct 11;14:1168951. doi: 10.3389/fpsyg.2023.1168951 (PMC10598845; doi:10.3389/fpsyg.2023.1168951)
Supplement: Supplementary file 1 [file Table_1.docx]

**Appendix**

**Nutrition Label Use Scale**

| Guidance note: Below are important questions related to nutrition label use. Five numbers are provided for each question; please circle the number you think most closely matches your feeling. | | | | | |
| --- | --- | --- | --- | --- | --- |
| Items | Strongly Disagree | Disagree | Neither agree nor disagree | Agree | Strongly Agree |
| Attitude |  |  |  |  |  |
| 1 Nutrition labels are accurate and reliable | 1 | 2 | 3 | 4 | 5 |
| 2 Nutrition label use can help me obtain important nutrition information of food | 1 | 2 | 3 | 4 | 5 |
| 3 Nutrition label use can help me choose healthy food | 1 | 2 | 3 | 4 | 5 |
| 4 Nutrition label use can help me compare food | 1 | 2 | 3 | 4 | 5 |
| 5 Nutrition label use can help me avoid eating foods with high fat and salt | 1 | 2 | 3 | 4 | 5 |
| 6 Nutrition label use can help prevent disease | 1 | 2 | 3 | 4 | 5 |
| 7 Nutrition label use can help in the treatment of diseases such as coronary heart disease | 1 | 2 | 3 | 4 | 5 |
| 8Nutrition label use promotes healthy eating habits | 1 | 2 | 3 | 4 | 5 |
| 9 Nutrition label use is convenient | 1 | 2 | 3 | 4 | 5 |
| 10 Nutrition label use is necessary | 1 | 2 | 3 | 4 | 5 |
| Subjective Norm |  |  |  |  |  |
| 11 My family expect me to use nutrition labels | 1 | 2 | 3 | 4 | 5 |
| 12 Doctors or nurses expect me to use nutrition labels | 1 | 2 | 3 | 4 | 5 |
| 13 My friends expect me to use nutrition labels | 1 | 2 | 3 | 4 | 5 |
| 14 Mass media(TV, newspapers, books or mobile phones) expect us to use nutrition labels | 1 | 2 | 3 | 4 | 5 |
| 15 Fellow sufferers expect me to use nutrition labels | 1 | 2 | 3 | 4 | 5 |
| 16 Community workers expect me to use nutrition labels | 1 | 2 | 3 | 4 | 5 |
| Perceived Behavioral Control |  |  |  |  |  |
| 17 The information of ingredients on nutrition labels encourage me to use it | 1 | 2 | 3 | 4 | 5 |
| 18 The intuitiveness of the nutrition label encourage me to use it | 1 | 2 | 3 | 4 | 5 |
| 19 The quest to be healthy motivates me to use nutrition labels | 1 | 2 | 3 | 4 | 5 |
| *20 Nutrition labels on the back of the food package discourage me from using it | 1 | 2 | 3 | 4 | 5 |
| *21 The numerical expression of the nutrition label discourage me from using it | 1 | 2 | 3 | 4 | 5 |
| *22The small font size of the nutrition labels discourage me from using it | 1 | 2 | 3 | 4 | 5 |
| *23Lack of knowledge about nutrition labels discourage me to use nutrition labels | 1 | 2 | 3 | 4 | 5 |
| *24 Impulse eating habits discourage me to use nutrition labels | 1 | 2 | 3 | 4 | 5 |
| *25 Time constraints discourage me to use nutrition labels | 1 | 2 | 3 | 4 | 5 |
| *26 Being with someone else restricts my use of nutrition labels | 1 | 2 | 3 | 4 | 5 |
| *27 Preference for specific foods restricts my use of nutrition labels | 1 | 2 | 3 | 4 | 5 |
| *28 Family financial strain restricts my use of nutrition labels | 1 | 2 | 3 | 4 | 5 |
| Intention |  |  |  |  |  |
| 29 I intent to learn about nutrition labels | 1 | 2 | 3 | 4 | 5 |
| 30 I intent to use nutrition labels | 1 | 2 | 3 | 4 | 5 |
| 31 I intent to use nutrition labels as often as possible | 1 | 2 | 3 | 4 | 5 |
| 32 I intent to select food according to the nutrition labels | 1 | 2 | 3 | 4 | 5 |
| 33 I intent to recommend others to use nutrition labels | 1 | 2 | 3 | 4 | 5 |

Note: *are the reverse items

Score of reverse items = (6-number of answer option)

Score for remaining items = number of answer option
